# Supplementary material for: Cytoskeleton stability is essential for the integrity of the cerebellum and its motor- and affective-related behaviors
Source: Sci Rep. 2018 Feb 15;8:3072. doi: 10.1038/s41598-018-21470-2 (PMC5814431; doi:10.1038/s41598-018-21470-2)
Supplement: Supplementary file 1 — Supplementary information [file 41598_2018_21470_MOESM1_ESM.docx]

**Cytoskeleton stability is essential for the integrity of the cerebellum and its motor- and affective-related behaviors**

Rodrigo Muñoz-Castañeda ^1,2^, David Díaz ^1,2^, Leticia Peris ^3,4^, Annie Andrieux ^3,4,5^, Christophe Bosc ^3,4^, José M. Muñoz-Castañeda ^6^, Carsten Janke ^7,8,9^, José R. Alonso^1,2,10^, Marie-Jo Moutin^3,4^, Eduardo Weruaga^1,2^

1. Laboratory of neural plasticity and neurorepair. Institute for Neurosciences of Castile and Leon (INCyL), University of Salamanca. E-37007 Salamanca, Spain.
2. Institute for Biomedical Research of Salamanca (IBSAL). E-37007 Salamanca, Spain.
3. Inserm, U1216, F-38000 Grenoble, France
4. Univ. Grenoble Alpes, Grenoble Institut des Neurosciences, GIN, F-38000 Grenoble, France, Université Grenoble Alpes, Grenoble Institut des Neurosciences, GIN, F-38000 Grenoble, France; Inserm, U1216, F-38000 Grenoble, France;
5. CEA, BIG-GPC, F-38000 Grenoble, France
6. Physics Department, Aeronautics Engineering School, Polytechnic University of Madrid. E-28040 Madrid, Spain
7. Institut Curie, F-91405 Orsay, France;
8. Paris Sciences et Lettres Research University, F-75005 Paris, France
9. Centre National de la Recherche Scientifique, UMR3348, F-91405 Orsay, France
10. Institute for Higher Research. University of Tarapaca. Arica, Chile.

**Corresponding author:** Dr. **Eduardo Weruaga**, INCyL, Universidad de Salamanca, C/ Pintor Fernando Gallego 1, E-37007, Salamanca, Spain; Phone: +34 923294500 Ext. 5324; Fax: + 34923294750; E-mail: ewp@usal.es.

**Abbreviated title:** The role of microtubules in cerebellar-related behavior

**Total word count** (Introduction + Results + Discussion): 3085 **Abstract**: 199

**Figures**: 7 **Number of pages**: 31 (including this)

**Conflict of interest**

The authors declare no competing financial interests.

**Acknowledgments**

This work was supported by the MINECO (SAF2016-79668-R; to EW and MTM2014-57129-C2-1-P to JMMC), the Regional Government of Castile and Leon (SA178U13 and SA030P17 to EW and RMC), the Centre for Regenerative Medicine and Cell Therapy of Castile and Leon (EW), the Samuel Solórzano Barruso Memorial Foundation (FS/3 2014 to DD), the Foltra Foundation (DD), INSERM, ARC (Association pour la Recherche sur le Cancer grant n°SFI20111204053 to MJM) , La Ligue contre le Cancer comités Savoie/Isère (MJM), ANR Blan07-2_187328 'TyrTIPs' (AA) and INCA PLBIO10-030 'TetraTips' (AA). The authors express their gratitude to E. J. Keck for the English language editing.

**ABSTRACT**

The cerebellum plays a key role in motor tasks, but its involvement in cognition is still being considered. Although there is an association of different psychiatric and cognitive disorders with cerebellar impairments, the lack of time-course studies has hindered the understanding of the involvement of cerebellum in cognitive and non-motor functions. Such association was here studied using the Purkinje Cell Degeneration mutant mouse, a model of selective and progressive cerebellar degeneration that lacks the cytosolic carboxypeptidase 1 (CCP1). The effects of the absence of this enzyme on the cerebellum of mutant mice were analyzed both *in vitro* and *in vivo*. These analyses were carried out longitudinally (throughout both the pre-neurodegenerative and neurodegenerative stages) and different motor and non-motor tests were performed. We demonstrate that the lack of CCP1 affects microtubule dynamics and flexibility, defects that contribute to the morphological alterations of the Purkinje cells (PCs), and to progressive cerebellar breakdown. Moreover, this degeneration led not only to motor defects but also to gradual cognitive impairments, directly related to the progression of cellular damage. Our findings confirm the cerebellar implication in non-motor tasks, where the formation of the healthy, typical PCs structure is necessary for normal cognitive and affective behavior.

**Keywords**: cerebellum, cognition, behavior, microtubules, polyglutamylation, Nna1/Ccp1, Purkinje cell degeneration

**INTRODUCTION**

There is a consensus regarding the key role of the cerebellum in motor tasks, but its involvement in cognitive and affective functions is not well known ^1,2^. Physiological and neuroimaging studies have shown cerebellar activation in non-motor tasks ^3–7^, and also some cerebellar impairments have been related to different psychiatric and neurodevelopmental disorders, such as schizophrenia, autism spectrum disorders or Niemann-Pick disease ^8–13^. The few animal studies so far performed indicate that the severity of the cerebellar abnormalities directly determine the extent to which cognition is affected ^14,15^. Thus, due to the lack of time-course studies it is not clear how the nature of cerebellar defects influences the performance of non-motor behaviors ^16^. Previous works have demonstrated the importance of both migration and morphogenesis of Purkinje cells (PCs) for correct cerebellar function ^17,18^, but the repercussion of progressive alterations of the cerebellar circuitry on the development of non-motor behaviors is not well documented.

Microtubules are essential for normal neuronal development and function; they change their structure and dynamics during cellular development and fate. In the brain, this molecular plasticity is necessary for neuronal migration, but also enables neurons to modify their morphology during development and to maintain this morphology as mature neurons ^17–19^. Microtubular alterations have been linked to different cognitive disorders, as well as to cerebellar defects ^13,20–23^. In this sense, microtubule dynamics has been proposed to be the basis of the cerebellar destruction in different animal models, such as the Purkinje Cell Degeneration (PCD) mutant mouse ^24,25^. However, it is not known how microtubule dynamics shape cerebellar structure and function along postnatal development.

In this context, the PCD mouse provides a good model to study the impact of cerebellar disorders on social and cognitive disorders. The PCD mouse suffers a mutation in the *Nna1* gene, which encodes cytosolic carboxypeptidase 1 (CCP1), an enzyme responsible for the de-polyglutamylation of microtubules ^24^. This mutation induces the degeneration of the Purkinje cells (PCs) of the cerebellum, but its effect on microtubule dynamics and structure remains to be determined. The progression of the death of PCs has been previously characterized and divided into two different stages: 1) an initial stage with cytoplasmic and nuclear alterations (from postnatal day 15, P15, to P18) and 2) a neurodegenerative process where all PCs die (P18-P45), leading to an aberrant cerebellum without the main projecting neurons ^26–28^. Interestingly, the expression of *Nna1* gene increases in the normal cerebellum from P15 to P25 ^29^, just when Purkinje cells dendritic arbors are re-modeling ^30,31^. Thus, CCP1 seems to be closely related to Purkinje cells maturation. Furthermore, previous studies have demonstrated that no other neuronal populations are affected during cerebellar degeneration in the PCD mouse ^25,28,29^; therefore making it a good model for studying the progression of alterations in the cerebellar structure and its influence on motor, cognitive and social behaviors. Also, since neuronal degeneration of the PCD mouse is known to be associated with microtubule defects ^32^, this model allows us to understand, first, the effect of polyglutamylation on microtubules dynamics and structure and, second, how these alterations affect postnatal cognitive and social behaviors along the neurodegenerative process. Thus, in order to study the impact of the lack of the CCP1 enzyme on behavioral development and cerebellar histology we used this well-established model ^25,33,34^. For the study of microtubules dynamics and morphology we generated a CCP1 KO mouse line that mimics the PCD mutation ^32^.

The main goal of this work is to understand the influence of microtubule dynamics and structure on cerebellar development and function, especially in Purkinje cells. To do this, we first studied the effects the lack of a functional CCP1 enzyme has on microtubule dynamics and structure in a generated CCP1 KO mouse line. We then analyzed the impact of microtubule alterations on PCs morphology and on general cerebellar structure in the original PCD mouse, which has a spontaneous deletion of the CCP1 enzyme. Finally, we analyzed in these PCD mice the effect of progressive cerebellum degeneration on motor, cognitive, and social processes by means of an animal model lacking the CCP1 enzyme.

**RESULTS**

***Effect of* pcd *mutation on microtubule dynamics and structure***

The lack of CCP1 induces an over-polyglutamylation of microtubules in the brain ^24^, which may influence their interaction with different proteins including severing and motor proteins ^35,36^. However, the effect on microtubule dynamics is poorly understood. To address this, we studied the microtubules of mouse embryonic fibroblasts (MEFs) lacking the CCP1 enzyme, which we named PCD microtubules (**Fig. 1**). Results showed (**Table 1**) that “growing rate” (*p*=0.022) and “catastrophe frequency” (*p*=0.012) were significantly increased in the PCD microtubules (**Fig. 1A-C**). Regarding microtubule curvature, PCD microtubules were more curved than the controls (*p*=0.003**; Fig. 1D-F**). In parallel, the curvature of trajectories was also increased in PCD microtubules (*p*=0.024**; Fig. 1G-I**). The lack of the CCP1 enzyme increased the instability of microtubules and influenced microtubule flexibility, thus affecting their trajectories.

***Morphological changes in the main dendrite of PCs begin during the pre-neurodegeneration***

Microtubules are essential for maintaining neuronal shape ^13,37^. Therefore, to understand the impact of these microtubular defects, we analyzed the morphology of PCs (**Fig. 2A;** **Table 2**) during different stages of PCD postnatal development: 1) P7, where no defects have been previously described, 2) P15 and P17, to characterize the pre-neurodegenerative stage and 3) P22 and P30, to characterize the neurodegenerative stage, when PCs disappear. Results showed that morphological changes of the main dendrite length (P7, *p*=0.483; P15, *p*=0.393; P17, *p*=0.028; P22, *p*=0.043; P30, *p*=0.027) and width (P7, *p*=0.379; P15, *p*=0.007; P17, *p*=0.017; P22, *p*=0.695; P30, *p*=0.020) began during pre-neurodegeneration (**Fig. 2B-C**), both parameters decreased in PCD mice. By contrast, a reduction of the dendritic arbor length - molecular layer thickness - (P7, *p*=0.361; P15, *p*=0.064; P17, *p*=0.704; P22, *p*=0.041; P30, *p*=0.030) and of the soma size (P7, *p*=0.484; P15, *p*=0.466; P17, *p*=0.085; P22, *p*=0.008; P30, *p*=0.001) was observed later, during the neurodegenerative stage (**Fig. 2D-E**). Hence, microtubule instability may induce morphological alterations of the PCs throughout late postnatal stages of cerebellar development, before the beginning of neurodegeneration.

***TUNEL analysis revealed general neuronal death in the cerebellum of PCD mice***

The *Nna1/Ccp1* gene mutation is known to affect all neuronal populations of the cerebellum ^24,25,27^. However, apart from PC loss, the progression of cell death of other cerebellar neuronal populations of the PCD mouse has not been previously characterized. Therefore, we monitored the death of the three main neuronal populations of the cerebellum: the molecular layer cells, the PCs and the granular layer cells (**Fig. 3**). Results confirmed that the PCs began to die in the PCD mouse from P22 and onwards, resulting in a process of cerebellar degeneration (P7, *p*=0.276; P15, *p*=0.073; P17, *p*=0.444; P22, *p*=0.004; P30, *p*=0.012; **Fig. 3D**. We also found an increase in cellular death during the same period in both the molecular (P7, *p*=0.168; P15, *p*=0.411; P17, *p*=0.302; P22, *p*<0.001; P30, *p*=0.010; **Fig. 3C**) and granular layers (P7, *p*=0.581; P15, *p*=0.960; P17, *p*=0.346; P22, *p*=0.029; P30, *p*<0.001; **Fig. 3E**). The neuronal nature of apoptotic cells in the three cerebellar layers was confirmed using TUNEL/NeuN and TUNEL/PV immunofluorescence colocalization (**Fig. 3A-B**). In summary, the initial symptoms of the breakdown of cerebellar structure are seen in the main dendrite of PCs. Following on, the morphological defects extend to the whole neuron, altering both the general dendritic arbor and soma. At the same time, there is a progressive and general neuronal loss in the other two layers resulting in a general breakdown of the cerebellum.

***Motor coordination is only affected during the neurodegeneration***

Data from the rota-rod test (**Fig. 4A-B**) showed that motor performance of the PCD mouse was not affected during pre-neurodegeneration (P15, *p*=0.677 and P17, *p*=0.398; **Fig. 4B**), but impaired during neurodegeneration; i.e. at P22 (*p*<0.001) and P30 (*p*<0.001; **Fig. 4B**). Thus, motor coordination is affected only during PC loss, but not prior to this.

***Home-cage behavior is altered in PCD mice before PC* *death***

Results of the home-cage behavior test showed no differences between genotypes regarding the time spent displacing, during any of the developmental stages, which indicated that the *pcd* mutation did not affect general movement (**Fig. 4D**). Concerning grooming time, no differences were found during pre-neurodegeneration (**Fig. 4E**), but it did decrease during neurodegeneration in the PCD mice (P15, *p*=0.444; P17, *p*=0.535; P22, *p=*0.005; P30, *p*<0.001). Interestingly, environmental exploratory behavior - rearings - was also reduced in PCD mice compared to the controls during both pre- (P15, *p*=0.042; P17, *p*<0.001) and neurodegeneration (P22, *p*=0.985; P30, *p*=0.005; **Fig. 4F**). Surprisingly, at P22 no changes were found in this exploratory behavior of PCD mice.

***Long-term memory is only affected in late neurodegenerative stages***

The results of the novel object recognition test showed that wild-type (WT) mice at all ages spent a large percentage of time exploring new rather than familiar objects (**Fig. 5**; P17, *p*=0.016; P22, *p*=0.004; P30, *p*=0.001), indicating a normal memory recognition. Similarly, PCD mice also presented the same behavior at P17 (*p*=0.016) and P22 (*p*=0.010). However, at P30 no differences were detected in the PCD mice regarding the percentage of time spent exploring both types of objects (*p*=0.497), suggesting a deficit in object memory recognition at this age.

The number of visits to both objects at P30 was further analyzed to avoid possible biases derived from motor alterations in PCD mice. Results did not show differences between the genotypes (**Fig. 5E**), thus discarding any effects of ataxia in PCD mice, with respect to moving within the plastic box and exploring the objects, and validating the previous result.

***Social preference is affected in PCD mice because of cerebellar pre-neurodegeneration***

The results of the social preference test revealed that WT mice at all ages spent a larger percentage of time exploring the chamber containing the animal rather than the chamber containing the object (P15, *p*=0.028; P17, *p*=0.042; P22, *p*=0.001; P30, *p*<0.001; **Fig. 6**), indicating a preference for social contact. However, PCD mice spent the same percentage of time exploring both chambers at all ages (P15, *p*=0.305; P17, *p*=0.328; P22, *p*=0.272; P30, *p*=0.812; **Fig. 6**). Thus, the pre-neurodegenerative changes observed prior to the breakdown of cerebellar structure seem to be enough to induce social deficits.

In conclusion, the behavioral analysis of PCD mice strongly suggests a close relationship between cerebellar function and cognition. Moreover, the progression of the cerebellar alterations increasingly impaired the performance of cognitive and affective tasks.

**DISCUSSION**

One of the main obstacles to establishing a relationship between the cerebellum and cognitive and social processes is the lack of studies analyzing the impact of progressive cerebellar defects on non-motor behavior. Also, this association has only been studied over short time frames. Here, to allow for a better understanding of this relationship, our results will be discussed chronologically based on their appearance throughout postnatal development in normal and PCD mice. The relevant order includes the first week of postnatal development (P7), then during the pre-neurodegenerative stage (P15-P17), and finally during the neurodegenerative stage (P22-P30). Thus, a temporal pattern is presented, characterizing the microtubule alterations observed in the PCD mouse, the progression of the cerebellar defects, and the influence of these alterations on motor and non-motor behaviors.

To understand the importance of the CCP1 enzyme in cerebellar development, the effect of the lack of this enzyme on microtubule dynamics and the structure of fibroblasts was initially studied, as the *in vitro* model available was able to provide insight into the structure and dynamics of this cellular component. While previous results have demonstrated that the lack of CCP1 enzyme increases microtubule polyglutamylation ^24^, here it is shown that the lack of this de-polyglutamylase affects microtubule stability and flexibility. Microtubules are more unstable at the growth cones, which consequently increases their catastrophe frequency, an essential process for normal neuronal development ^38,39^. Additionally, certain factors related to neuronal migration and development preferentially bind to curved microtubules ^40–43^. Therefore, microtubule dynamics and curvature seem to have a different pattern during neuronal development and maturation, being more dynamic and curved during development and stable and rigid during maturation. The microtubules of PCD mice are likely very similar to those found in developing neurons, in terms of dynamics and structure. In WT mice, CCP1 expression increases at P15 in the cerebellum ^29^, that is, at the beginning of the pre-neurodegeneration stage of the PCD mouse ^26,29^. Precisely, during this short time frame, the growth of the dendritic arbor of PCs is completed and the process of maturation and remodeling takes place ^44,45^. Thus, microtubule polyglutamylation seems to be essential during cerebellar development, and a reduction of this post-translational modification seems to be necessary at the end of neuronal growth.

Then, the microtubule over-polyglutamylation occurring in the PCD mouse triggers the pre-neurodegeneration and the loss of the PCs from P18 and onwards ^25,26,29^. Conversely, at P7 no differences were observed in the morphology of the PCs between genotypes, the general structure of the cerebellum was apparently unaffected. Since neuronal migration and growth occur in the normal cerebellar cortex during this first postnatal week ^46^, it appears that the *pcd* mutation (i.e., microtubule over-polyglutamylation) does not affect the initial perinatal stages of cerebellar cortex formation. CCP1 activity is extremely low during the first two postnatal weeks (it significantly increases at P15) ^29^ thus, our data support the idea that microtubule polyglutamylation is essential during early cerebellar development, but deleterious for establishing a mature pattern. Therefore, the fine-tuning of microtubule polyglutamylation has a major role in cerebellar postnatal development.

In order to understand the influence of the cerebellum on cognitive and social behavior, different authors have analyzed the effects of different cerebellar alterations in animal models ^14,15^. However, to our knowledge, there are no studies that have attempted to analyze progressive cerebellar alterations throughout postnatal development to understand the normal cerebellar function.

Morphological defects in the PCs of different psychiatric and neurodevelopmental disorders have been recently described ^47–49^, but the direct effect of alterations in the morphology of PCs on non-motor behavior is not known. We demonstrate that prior to the breakdown of cerebellar structure (during pre-neurodegeneration, when no motor defects were detected), morphological alterations in the main dendrite of PCs and social and environmental exploratory behavioral disabilities occur simultaneously (both at P15 and P17). In psychiatric and neurodevelopmental disorders, such as autism or schizophrenia that can often exhibit severe symptoms of social disability, have been related to cerebellar alterations ^8–12,15^. Furthermore, PC loss is the most common anatomopathological change found in autistic patients ^47^, and even in those cases where no loss of PCs was observed, morphological defects were still found ^48^. Also, in accordance with our results, environmental exploratory defects have been previously associated with cerebellar alterations ^50–52^. A possible explanation for these impairments is that alterations in dendritic morphology influence the electrophysiological activity of neurons, including the PCs ^53,54^. More precisely, defects in the PCs axial resistance, which directly depends on dendritic length and width, disrupt the synaptic integration in PCs ^53^, as previously hypothesized ^55,56^. Hence, it is plausible to relate the progressive extinction of Purkinje cells in PCD mice to the progressive alteration of the behaviors analyzed (see below).

During the cerebellar degeneration that occurs in PCD mice, neuronal death takes place throughout the three cerebellar layers. Previous studies assumed that in the PCD animal, the loss of neuronal populations of the granular and molecular layers is residual and delayed in time, in comparison to the death of PCs ^25^; however, the progression of their death has never been characterized. Our results demonstrate that this neuronal loss progresses at the same time as the loss of PCs, indicating a simultaneous decline in all three layers in the cerebellum of the PCD mouse.

During neurodegeneration, the morphological alterations of the PCs worsen, with the most evident changes linked to the length and width of the main dendrite, the soma area and the dendritic arbor length. In parallel, the defects observed in social preference and environmental exploratory behavior also persist during this stage in the PCD mouse. Alterations in these behaviors have been described in both animal and human studies with cerebellar impairments ^8–10,57–59^. It was observed that these alterations may be due both to neuronal loss and to PC malfunction. Interestingly, we saw that ataxia did not bias the results of the cognitive/social tasks performed by the PCD mice, and during neurodegeneration grooming was found to be reduced. In contrast, this behavior has been previously shown to increase in animal models of specific PC loss ^15^, being more so in models presenting a general alteration of the cerebellum ^58^. Accordingly, during grooming behavior there is an increase in the activity of both granule cells ^60^ and PCs ^59^. Consequently, grooming may be affected differently depending on whether the cerebellar alterations affect the PCs, granule cells or both. Thus, the severity of cerebellar defects helps to understand the relationship between the cerebellum and grooming. Although grooming is a very complex motor task, we cannot discard the possibility that the motor defects caused by cerebellar alterations have influenced the observed behavioral changes.

Interestingly, general changes in cerebellar structure in the PCD mouse, which disrupt recognition memory, only occurred at P30 when most of the PCs are lost. The cerebellum has been associated with procedural learning and memory ^61,62^, and current neuroimaging studies have also described activation of the cerebellum during non-procedural memory tasks ^63–65^. These works, however, did not study the impact of the progression of the breakdown of cerebellar structure on recognition memory. According to our results, cerebellar alterations lead to the impairment of recognition memory in PCD mice, and during neurodegeneration both the cerebellar and behavioral defects become worse. Therefore, these results suggest a correlation between the progression of the breakdown of the cerebellar structure and the severity of the defects related to performing cognitive and affective tasks. Then, a healthy, typical cerebellar structure is necessary to execute specific cognitive and affective tasks.

In conclusion, polyglutamylation affects microtubules dynamics and structure, and its modulation is essential throughout normal cerebellar development. During the first two postnatal weeks, when the PCs are still migrating and growing, microtubules instability is crucial. However, a pathologically extended instability of microtubules (triggered by the *pcd* mutation) affects the morphology and survival of PCs, which are vital for the correct functioning of the cerebellum. In this sense, cerebellum plays an important role in cognitive and affective functions, since the progressive breakdown of the cerebellar cortex in PCD mice is directly related to the increasing severity of cognitive and affective impairments.

**MATERIAL AND METHODS**

***Animals***

Both WT and PCD mice from the C57/DBA strain were used and purchased from The Jackson Laboratory, Maine, USA. For *in vitro* studies, MEFs were prepared from both WT and CCP1-KO embryos (see below). The embryos were separated into groups depending on their genotype (WT or PCD; n=3 embryos per group). For *in vivo* studies, animals were separated into groups depending on their genotype and age at the time of analysis: P7, P15, P17, P22 and P30 (n=4 per age and genotype) for the immunohistochemical analyses; and P15, P17, P22 and P30 (n=8-9 per genotype) for the behavioral analyses.

Since PCD and CCP1-KO animals are not fertile, heterozygous mice were mated, and the embryos or newborn mice were genotyped (see below and ^28^).

Animals were housed, handled and sacrificed per the guidelines established by European (2010/63/UE) and national legislations (Spanish RD53/2013 and Law 32/2007; French permit n°38 07 11). Every effort was made to minimize the number of animals used and their suffering. This study was approved by the Bioethics Committee of the University of Salamanca.

***Generation of CCP1 KO mice***

The conditional mutant mouse line for CCP1 (on exons 20 and 21) was established and generated at the Mouse Clinical Institute (MCI, Illkirch, France) to study microtubules dynamics and structure. The targeting vector was constructed as follows. The 5’ (4.5 kb), 3’ (1.4 kb) and inter-loxP (1.9) fragments were polymerase chain reaction (PCR) amplified and sequentially subcloned into an MCI proprietary vector containing the LoxP sites and a Neo cassette flanked by Flippase Recognition Target (FRT) sites (**Fig. 7**). The linearized construct was electroporated in 129S2/SvPas mouse embryonic stem (ES) cells. After selection, targeted clones were identified by PCR using external primers and further confirmed by Southern blot with 5’ and 3’ external probes. Two positive ES clones were injected into blastocysts, and derived male chimaeras gave germline transmission. The excision of the neomycin-resistance cassette was performed *in vivo* by breeding the chimeras with a Flp deleter line (C57BL/6N genetic background FLP under ACTB promoter). The Flp transgene was segregated by breeding the first germ line mice with a wild type C57BL/6N animal. For generation *ccp1* KO, *ccp1* floxed mice were crossed with transgenic mice expressing Cre recombinase under the control of a CMV promoter.

Genomic DNA isolated from mouse tail snip was analyzed by PCR. Mice were genotyped by PCR according to MCI protocols using GoTag polymerase (Promega) and 33 amplification cycles. The three primer pairs listed below were used to define the genotypes.

(1) Ef: GTACACAGACTTTTAGGCTAATCTC

Er: ACTTCCACATCTGCACTGTGGACAG

(2) Lf: CATAAGAACCAGGTTCATTCTGTCC

Lr: TAGCATCATGTTAAAACTCCCTCCT

(3) Lf: CATAAGAACCAGGTTCATTCTGTCC

Er: ACTTCCACATCTGCACTGTGGACAG

***Cell culture and transfection***

For *in vitro* analysis of the microtubules, MEFs from the CCP1 KO line were prepared as previously described ^66^. To visualize the microtubules, MEFs were transfected using the Nucleofector^TM^ Kits for MEFs (Amaxa Biosystems) with GFP-EB3 (to visualize microtubules (+)-ends; provided by N. Galjart, Erasmus Medical Center, Rotterdam, The Netherlands) and m-cherry α-tubulin (to visualize the entire microtubules; provided by F. Saudou, Curie Institute, Paris, France) plasmids.

***Video microscopy***

Video time-lapses of transfected MEFs were captured with an inverted microscope (Axiovert 200M; Carl Zeiss, Oberkochen, Germany) and a 100× NA 1.3 Plan-Neofluar oil objective controlled by MetaMorph software (MDS Analytical Technologies, Sunnyvale, CA, USA). Images were taken every three seconds for 5 minutes. All images were captured with a charge-coupled device camera (CoolSNAP HQ; Roper Scientific, Sarasota, Florida, USA).

***Microtubule dynamics and structure analysis***

For the analysis of the microtubule dynamics the plusTipTracker software ^67^ and the GFP-EB3 video time-lapse images were employed. We first adjusted the accuracy of the GFP-EB3 detection, and then analyzed the following parameters: growing and shrinking speed; growth and shrinking mean length; percentage of time in pause, growing and shrinking; distribution of microtubules based on their speed and length; catastrophe frequency and rescue frequency. These parameters were chosen based on previous studies ^68,69^. To ensure the accuracy of the analysis, we compared the growing speed data obtained both automatically and manually.

The microtubule curvature analysis was performed using the first image of each video time-lapse with m-cherry α-tubulin. Additionally, with the GFP-EB3 labeling experiment we obtained the maximum projection of the entire video time-lapse, allowing us to reconstruct the trajectories of the microtubules, whose curvature was also analyzed. Thus, the curvature of microtubules was analyzed both in a static way and within their trajectory of movement.

Curvature ($k$) was determined using the Mathematica software (Wolfram Research Europe, UK). After obtaining the equation of each microtubule with a spline cubic interpolation, the general formula of curvature ^70^ was employed to analyze each microtubule (equation 1):

$$k=\frac{x´y´´-y´x´´}{\left( {x´}^{2}+{y´}^{2} \right)^{\frac{3}{2}}}$$

Where (equations 2, 3, 4 and 5)

$x´=\frac{dx}{dt}$ , $x´´=\frac{d^{2}x}{{dt}^{2}}$ , $y´=\frac{dy}{dt}$ , $y´´=\frac{d^{2}y}{{dt}^{2}}$ ,

i.e., the first ($x', y'$) and second ($x'',y'$) derivatives at each point.

***Tissue extraction and preparation for in vivo studies***

To analyze the effect of the *pcd* mutation on the PCs morphology and the general cerebellar structure *in vivo*, animals were anesthetized and intracardially perfused with Somogyi's fixative without glutaraldehyde (5 ml/g body weight), as previously described ^26^Cerebella were sectioned sagittally at 30 µm thick using a freezing microtome (Leica Jung SM 2000, Nussloch, Germany), as previously described ^71–73^. The histological analyses were focused on the vermis using three sections per animal (separated by 180 µm each), with a total of five animals per genotype and age. All values represent the mean count of the three sections analyzed.

***Immunofluorescence***

Sections were incubated with the primary antisera at 4 ^o^C during 72 h, as previously described ^74^. The antibodies employed were mouse anti-calbindin (Cb-28k 1:1,000; Swant, Switzerland), mouse anti-parvalbumin (PV 1:1,000; Swant, Switzerland) and mouse anti-NeuN (1:8,000; Merck Millipore, Darmstadt, Germany). Appropriate secondary antibodies conjugated with Cy2 or Cy3 (1:500; Jackson Laboratories, West Grove, PA, USA) were used. Sections were counter-stained with DAPI (1:30,000; Sigma Aldrich) to identify the cell nuclei. Antibodies were selected based on their selectivity to identify different neuronal populations. Calbindin is a well-known calcium-binding protein that label the whole PCs ^46,75,76^, allowing us to analyze the PCs morphology. In addition, parvalbumin stains both PCs and interneurons in the molecular layer ^46^. Finally, NeuN was used to counterstain whole cerebellar nuclei during cell counting, ensuring no biases were made due to the lack of immunolabeling during degeneration.

***Terminal Deoxynucleotidyl Transferase-Mediated Fluorescein dUTP Nick-End Labeling (TUNEL) detection***

To study apoptosis throughout cerebellar postnatal development, the TUNEL technique was used as previously described ^29,74^. To determine the neuronal nature of apoptotic cells, the TUNEL was combined with immunohistochemistry against NeuN or PV. Finally, both TUNEL and immunohistochemistry were revealed using Cy2-streptavidin and Cy3-secondary antibodies, respectively.

***Morphological analysis of PCs***

Morphological analysis of the PCs was made using sections immunostained for Cb-28k from P7 to P30. To avoid possible biases, only those Purkinje cells containing the soma and a clear dendritic arbor were analyzed. Also, all Purkinje cells fitting this criterion were analyzed in three cerebellar sections per animal (separated by 180 µm each). The final value for each animal used to compare was the mean of all Purkinje cells analyzed in these three sections. The morphological parameters studied were selected in basis to its meaning for PCs function ^77,78^: 1) the length and width of the main dendrite; 2) the length of the dendritic arbor of the PCs (measured indirectly through the molecular layer thickness); and 3) the soma size. Morphological analyses were carried out with the Neurolucida (MBF Bioscience, Williston, Vermont, USA) and ImageJ (NIH, USA) software, as previously described ^77^.

***Behavioral analyses***

We characterized the effects of progressive cerebellar defects on motor, cognitive, and social processes. Behavioral analyses were performed at P15, P17, P22 and P30, encompassing both the pre-neurodegenerative (P15-P18) and neurodegenerative (P18-P30) stages. All devices employed were cleaned with 96% (v/v) ethanol before and after each session.

The *rota-rod test* was used to characterize motor coordination. It was performed as previously described ^79^, with an acceleration of 0.6 rpm/s, from 4 to 40 rpm, in 10 min.

The *home-cage behavior analysis* was used to characterize general behavior. Animals were placed each day in the home-cage for 10 min and allowed to freely explore. Afterwards, the animals’ behavior was observed for 10 min, during which time the following parameters were recorded manually: 1) grooming time (stereotyped behavior); 2) number of rearings (environmental exploratory behavior) and 3) time moving (general movement). Each animal was assigned to an individual home-cage to avoid the influence of odor from the other animals.

The *novel object recognition test* was used to analyze recognition memory. The test was performed in the same home-cage used described above to help the animals to become habituated. On the first day, at P15, animals were placed in the home-cage with two identical objects (A and A) for 10 min. Then, for each of the following sessions (at P17, P22 and P30) the animals were placed in the home-cage with: 1) the familiar object (A) and 2) a new object (B, C, and D, respectively for each of the different ages; see **Fig. 5**). The objects were placed in opposite corners of the home-cage, the time interacting with each object was measured and their ratio was calculated.

The *social preference test* was performed in a white Plexiglas® box (50 x 29 cm) divided into three chambers. The social preference test was performed as previously described ^80–82^. On each test day, a mouse could explore the box for 10 min. Then, the animal was removed from the box and a different mouse, of the same age and sex, was placed in one of the lateral chambers under a drilled pencil cup. On the other side of the box, an object was placed in the other lateral chamber, also under a drilled pencil cup. The original mouse was then returned to the box and the percentage of time spent exploring each lateral chamber and each pencil cup was analyzed.

***Statistical analysis***

For data analysis, the Student’s *t*-Test was performed. For histological analysis, both genotypes were compared for each age. For behavioral analysis, the parameters analyzed and the different confrontation variables are described in each behavioral procedure. All analyses were performed with the SPSS statistical package software (IBM, NY, USA).

**REFERENCES**

1. Strick, P. L., Dum, R. P. & Fiez, J. a. Cerebellum and nonmotor function. *Annu. Rev. Neurosci.* **32,** 413–34 (2009).

2. Ito, M. Control of mental activities by internal models in the cerebellum. *Nat. Rev. Neurosci.* **9,** 304–313 (2008).

3. Allen, G. & Courchesne, E. Differential effects of developmental cerebellar abnormality on cognitive and motor functions in the cerebellum: An fMRI study of autism. *Am. J. Psychiatry* **160,** 262–273 (2003).

4. Allen, G., Buxton, R. B., Wong, E. C. & Courchesne, E. Attentional activation of the cerebellum independent of motor involvement. *Science* **275,** 1940–1943 (1997).

5. Fatemi, S. H. *et al.* Consensus paper: Pathological role of the cerebellum in Autism. *Cerebellum* **11,** 777–807 (2012).

6. Allen, G., Müller, R. A. & Courchesne, E. Cerebellar function in autism: Functional magnetic resonance image activation during a simple motor task. *Biol. Psychiatry* **56,** 269–278 (2004).

7. Piven, J. *et al.* Magnetic resonance imaging in autism: measurement of the cerebellum, pons, and fourth ventricle. *Biol. Psychiatry* **31,** 491–504 (1992).

8. Joseph, R. M., Tager-Flusberg, H. & Lord, C. Cognitive profiles and social-communicative functioning in children with autism spectrum disorder. *J. Child Psychol. Psychiatry* **43,** 807–821 (2002).

9. Armstrong, D. D. Rett syndrome neuropathology review 2000. *Brain Dev.* **23,** S72–S76 (2001).

10. Bauman, M. L. & Kemper, T. L. Neuroanatomic observations of the brain in autism: A review and future directions. *Int. J. Dev. Neurosci.* **23,** 183–187 (2005).

11. Walkley, S. U. & Suzuki, K. Consequences of NPC1 and NPC2 loss of function in mammalian neurons. *Biochim. Biophys. Acta* **1685,** 48–62 (2004).

12. Andreasen, N. C. & Pierson, R. The Role of the Cerebellum in Schizophrenia. *Biol. Psychiatry* **64,** 81–88 (2008).

13. Baas, P. W., Rao, A. N., Matamoros, A. J. & Leo, L. Stability properties of neuronal microtubules. *Cytoskeleton* **73**,442-460 (2016).

14. Galliano, E. *et al.* Synaptic transmission and plasticity at inputs to murine cerebellar Purkinje cells are largely dispensable for standard nonmotor tasks. *J. Neurosci.* **33,** 12599–618 (2013).

15. Tsai, P. T. *et al.* Autistic-like behaviour and cerebellar dysfunction in Purkinje cell Tsc1 mutant mice. *Nature* **488,** 647–651 (2012).

16. Brossard-Racine, M., du Plessis, A. & Limperopoulos, C. Developmental Cerebellar Cognitive Affective Syndrome in Ex-preterm Survivors Following Cerebellar Injury. *Cerebellum* **14,** 151–164 (2015).

17. Faivre, C., Legrand, C. & Rabié, A. The microtubular apparatus of cerebellar purkinje cell dendrites during postnatal development of the rat: The density and cold-stability of microtubules increase with age and are sensitive to thyroid hormone deficiency. *Int. J. Dev. Neurosci.* **3,** 559–565 (1985).

18. Kim, J. *et al.* Dendritic planarity of Purkinje cells is independent of Reelin signaling. *Brain Struct. Funct.* **220,** 2263–2273 (2014).

19. Liu, Y., Lee, J. W. & Ackerman, S. L. Mutations in the Microtubule-Associated Protein 1A (Map1a) Gene Cause Purkinje Cell Degeneration. *J. Neurosci.* **35,** 4587 LP-4598 (2015).

20. Andrieux, A. *et al.* The suppression of brain cold-stable microtubules in mice induces synaptic defects associated with neuroleptic-sensitive behavioral disorders. *Genes Dev.* **16,** 2350–2364 (2002).

21. Maussion, G. *et al.* Convergent evidence identifying MAP/microtubule affinity-regulating kinase 1 (MARK1) as a susceptibility gene for autism. *Hum. Mol. Genet.* **17,** 2541–2551 (2008).

22. Brown, A. S. *et al.* Increased stability of microtubules in cultured olfactory neuroepithelial cells from individuals with schizophrenia. *Prog. Neuro-Psychopharmacology Biol. Psychiatry* **48,** 252–258 (2014).

23. Cartelli, D. & Cappelletti, G. Microtubule Destabilization Paves the Way to Parkinson’s Disease. *Mol. Neurobiol.* **54**, 6762–6774 (2016).

24. Rogowski, K. *et al.* A family of protein-deglutamylating enzymes associated with neurodegeneration. *Cell* **143,** 564–578 (2010).

25. Wang, T. & Morgan, J. I. The Purkinje cell degeneration (pcd) mouse: An unexpected molecular link between neuronal degeneration and regeneration. *Brain Res.* **1140,** 26–40 (2007).

26. Baltanás, F. C. *et al.* Nucleolar disruption and cajal body disassembly are nuclear hallmarks of DNA damage-induced neurodegeneration in Purkinje cells. *Brain Pathol.* **21,** 374–388 (2011).

27. Baltanás, F. C. *et al.* Purkinje Cell Degeneration in pcd Mice Reveals Large Scale Chromatin Reorganization and Gene Silencing Linked to Defective DNA Repair. *J. Biol. Chem.* **286,** 28287–28302 (2011).

28. Valero, J., Berciano, M. T., Weruaga, E., Lafarga, M. & Alonso, J. R. Pre-neurodegeneration of mitral cells in the pcd mutant mouse is associated with DNA damage, transcriptional repression, and reorganization of nuclear speckles and Cajal bodies. *Mol. Cell. Neurosci.* **33,** 283–295 (2006).

29. Baltanás, F. C. *et al.* Differential glial activation during the degeneration of Purkinje cells and mitral cells in the PCD mutant mice. *Glia* **61,** 254–72 (2013).

30. Hashimoto, K. & Kano, M. Postnatal development and synapse elimination of climbing fiber to Purkinje cell projection in the cerebellum. *Neurosci. Res.* **53,** 221–228 (2005).

31. Nedelescu, H. & Abdelhack, M. Comparative morphology of dendritic arbors in populations of purkinje cells in mouse sulcus and apex. *Neural Plast.* **2013,** 948587 (2013).

32. Bosch Grau, M. *et al.* Alterations in the balance of tubulin glycylation and glutamylation in photoreceptors leads to retinal degeneration. *J. Cell Sci.* **130,** 938-949 (2017).

33. Ghetti, B., Triarhou, L. C., Alyea, C. J., Dlouhy, S. R. & Karn, R. C. Unique cerebellar phenotype combining granule and Purkinje cell loss: morphological evidence for weaver* pcd double mutant mice. *J. Neurocytol.* **20,** 27–38 (1991).

34. Mullen, R. J., Eicher, E. M. & Sidman, R. L. Purkinje cell degeneration, a new neurological mutation in the mouse. *Proc. Natl. Acad. Sci. U. S. A.* **73,** 208–212 (1976).

35. Lacroix, B. *et al.* Tubulin polyglutamylation stimulates spastin-mediated microtubule severing. *J. Cell Biol.* **189,** 945–54 (2010).

36. Sirajuddin, M., Rice, L. M. & Vale, R. D. Regulation of microtubule motors by tubulin isotypes and post-translational modifications. *Nat. Cell Biol.* **16,** 335–44 (2014).

37. Sakakibara, A., Ando, R., Sapir, T. & Tanaka, T. Microtubule dynamics in neuronal morphogenesis. *Open Biol.* **3,** 130061 (2013).

38. Dent, E. W., Callaway, J. L., Szebenyi, G., Baas, P. W. & Kalil, K. Reorganization and movement of microtubules in axonal growth cones and developing interstitial branches. *J. Neurosci.* **19,** 8894–8908 (1999).

39. Dubey, J., Ratnakaran, N. & Koushika, S. P. Neurodegeneration and microtubule dynamics: death by a thousand cuts. *Front. Cell. Neurosci.* **9,** 343 (2015).

40. Gleeson, J. G., Peter T, L., Flanagan, L. A. & Walsh, C. A. Doublecortin is a microtubule-associated protein and is expressed widely by migrating neurons. *Neuron* **23,** 257–271 (1999).

41. Bechstedt, S. & Brouhard, G. J. Doublecortin Recognizes the 13-Protofilament Microtubule Cooperatively and Tracks Microtubule Ends. *Dev. Cell* **23,** 181–192 (2012).

42. Fu, X. *et al.* Doublecortin (Dcx) family proteins regulate filamentous actin structure in developing neurons. *J. Neurosci.* **33,** 709–21 (2013).

43. Bechstedt, S., Lu, K. & Brouhard, G. J. Doublecortin Recognizes the Longitudinal Curvature of the Microtubule End and Lattice. *Curr. Biol.* **24,** 2366–2375 (2015).

44. Kaneko, M. *et al.* Remodeling of monoplanar Purkinje cell dendrites during cerebellar circuit formation. *PLoS One* **6,** e20108 (2011).

45. Kawaguchi, K., Habara, T., Terashima, T. & Kikkawa, S. GABA modulates development of cerebellar Purkinje cell dendrites under control of endocannabinoid signaling. *J. Neurochem.* **114,** 627–638 (2010).

46. White, J. J. & Sillitoe, R. V. Development of the cerebellum: from gene expression patterns to circuit maps. *Wiley Interdiscip. Rev. Dev. Biol.* **2,** 149–64 (2013).

47. Sudarov, A. Defining the role of cerebellar purkinje cells in autism spectrum disorders. *Cerebellum* **12,** 950–5 (2013).

48. Fatemi, S. H. *et al.* Purkinje cell size is reduced in cerebellum of patients with autism. *Cell. Mol. Neurobiol.* **22,** 171–175 (2002).

49. Mavroudis, I. A. *et al.* Dendritic and spinal pathology of the purkinje cells from the human cerebellar vermis in alzheimer’s disease. *Psychiatr. Danub.* **25,** 221–226 (2013).

50. Dahhaoui, M., Caston, J., Lannou, J. & Avenel, S. Role of the cerebellum in habituation exploration behavior in the rat. *Physiol. Behav.* **52,** 339–344 (1992).

51. Pierce, K. & Courchesne, E. Evidence for a cerebellar role in reduced exploration and stereotyped behavior in autism. *Biol. Psychiatry* **49,** 655–664 (2001).

52. Caston, J., Chianale, C., Delhaye-Bouchaud, N. & Mariani, J. Role of the cerebellum in exploration behavior. *Brain Res.* **808,** 232–7 (1998).

53. Bekkers, J. M. Changes in dendritic axial resistance alter synaptic integration in cerebellar Purkinje cells. *Biophys. J.* **100,** 1198–1206 (2011).

54. Krichmar, J. L., Nasuto, S. J., Scorcioni, R., Washington, S. D. & Ascoli, G. A. Effects of dendritic morphology on CA3 pyramidal cell electrophysiology: A simulation study. *Brain Res.* **941,** 11–28 (2002).

55. Schmahmann, J. D. The role of the cerebellum in affect and psychosis. *J. Neurolinguistics* **13,** 189–214 (2000).

56. Schmahmann, J. D. The cerebellar cognitive affective syndrome: clinical correlations of the dysmetria of thought hypothesis. *Int. Rev. Psychiatry* **13,** 313–322 (2001).

57. Ismail, M. M. T. *et al.* Studying Autism Spectrum Disorder with Structural and Diffusion Magnetic Resonance Imaging: A Survey. *Front. Hum. Neurosci.* **10,** 211 (2016).

58. Strazielle, C. & Lalonde, R. Grooming in Lurcher mutant mice. *Physiol. Behav.* **64,** 57–61 (1998).

59. Ghosh, K. K. *et al.* Miniaturized integration of a fluorescence microscope. *Nat. Methods* **8,** 871–878 (2011).

60. Hartmann, M. J. & Bower, J. M. Tactile responses in the granule cell layer of cerebellar folium crus IIa of freely behaving rats. *J. Neurosci.* **21,** 3549–3563 (2001).

61. Molinari, M. *et al.* Cerebellum and procedural learning: Evidence from focal cerebellar lesions. *Brain* **120,** 1753–1762 (1997).

62. Torriero, S. *et al.* Changes in cerebello-motor connectivity during procedural learning by actual execution and observation. *J. Cogn. Neurosci.* **23,** 338–48 (2011).

63. Weis, S., Klaver, P., Reul, J., Elger, C. E. & Fernández, G. Temporal and Cerebellar Brain Regions that Support both Declarative Memory Formation and Retrieval. *Cereb. Cortex* **14,** 256–267 (2004).

64. Cabeza, R., Kapur, S., Craik, F. I. M. & McIntosh, A. R. Functional neuroanatomy of recall and recognition: A PET study of episodic memory. *J. Cogn. Neurosci.* **9,** 254–265 (1997).

65. Buckner, R. L. The cerebellum and cognitive function: 25 years of insight from anatomy and neuroimaging. *Neuron* **80,** 807–815 (2013).

66. Erck, C. *et al.* A vital role of tubulin-tyrosine-ligase for neuronal organization. *Proc. Natl. Acad. Sci. U. S. A.* **102,** 7853–7858 (2005).

67. Matov, A. *et al.* Analysis of microtubule dynamic instability using a plus-end growth marker. *Nat. Methods* **7,** 761–768 (2010).

68. Peris, L. *et al.* Motor-dependent microtubule disassembly driven by tubulin tyrosination. *J. Cell Biol.* **185,** 1159–1166 (2009).

69. Applegate, K. T. *et al.* PlusTipTracker: Quantitative image analysis software for the measurement of microtubule dynamics. *J. Struct. Biol.* **176,** 168–184 (2011).

70. Goetz, A. *Introduction to differential geometry*. Addison Wesley Pub. Co, 1970. New York, USA. ISBN 10: 0201024314.

71. Zhao, Z. *et al.* Genetic Ablation of PLA2G6 in Mice Leads to Cerebellar Atrophy Characterized by Purkinje Cell Loss and Glial Cell Activation. *PLoS One* **6,** e26991 (2011).

72. Shimizu-Albergine, M. *et al.* Individual cerebellar Purkinje cells express different cGMP phosphodiesterases (PDEs): in vivo phosphorylation of cGMP-specific PDE (PDE5) as an indicator of cGMP-dependent protein kinase (PKG) activation. *J. Neurosci.* **23,** 6452–9 (2003).

73. Kaemmerer, W. F. *et al.* In vivo transduction of cerebellar Purkinje cells using adeno-associated virus vectors. *Mol. Ther.* **2,** 446–57 (2000).

74. Díaz, D., Recio, J. S., Weruaga, E. & Alonso, J. R. Mild cerebellar neurodegeneration of aged heterozygous PCD mice increases cell fusion of Purkinje and bone marrow-derived cells. *Cell Transplant.* **21,** 1595–1602 (2012).

75. Hoebeek, F. E., Khosrovani, S., Witter, L. & De Zeeuw, C. I. Purkinje cell input to cerebellar nuclei in tottering: ultrastructure and physiology. *Cerebellum* **7,** 547–58 (2008).

76. Sillitoe, R. V & Joyner, A. L. Morphology, molecular codes, and circuitry produce the three-dimensional complexity of the cerebellum. *Annu. Rev. Cell Dev. Biol.* **23,** 549–77 (2007).

77. Donald, S. *et al.* P-Rex2 regulates Purkinje cell dendrite morphology and motor coordination. *Proc. Natl. Acad. Sci. U. S. A.* **105,** 4483–4488 (2008).

78. Fujishima, K., Horie, R., Mochizuki, A. & Kengaku, M. Principles of branch dynamics governing shape characteristics of cerebellar Purkinje cell dendrites. *Development* **139,** 3442–3455 (2012).

79. Muñoz-Castañeda, R., Díaz, D., Ávila-Zarza, C. A., Alonso, J. R. & Weruaga, E. Sex-influence of nicotine and nitric oxide on motor coordination and anxiety-related neurophysiological responses. *Psychopharmacology (Berl).* **231,** 695–706 (2014).

80. Crawley, J. N. Designing mouse behavioral tasks relevant to autistic-like behaviors. *Ment. Retard. Dev. Disabil. Res. Rev.* **10,** 248–258 (2004).

81. Panksepp, J. B. & Lahvis, G. P. Social reward among juvenile mice. *Genes, Brain Behav.* **6,** 661–671 (2007).

82. Silverman, J. L., Yang, M., Lord, C. & Crawley, J. N. Behavioural phenotyping assays for mouse models of autism. *Nat. Rev. Neurosci.* **11,** 490–502 (2010).

**Author contribution statement**

RM-C, DD, M-JM, AA and EW designed the experiments; CJ designed the conditional mutant mouse line for CCP1; RM-C, M-JM, LP and CB did the *in vitro* experiments; RM-C did *in vivo* experiments and behavioral tests; RM-C, DD and EW performed the histological analysis; RM-C and JMM-C performed the mathematical analyses of microtubule curvature; RM-C did statistical analyses; RM-C and DD prepared the figures; RM-C, DD, M-JM, AA, JRA and EW wrote the manuscript, and all authors revised the manuscript.

**Figure 1:** Effect of *pcd* mutation on microtubule dynamics and structure (mean±SEM). A and B, manual and automatic analyses of microtubules growth and shrinking rates; in both analysis PCD microtubules showed an increase in growing rate. C, catastrophe frequency analysis; an increase in the frequency of catastrophes in PCD microtubules is demonstrated. D-F, analysis of WT (D) and PCD (E) microtubules curvature and its graphical representation (F); an increase in the curvature of PCD microtubules can be observed. G-I, Analysis of WT (G) and PCD (H) microtubule trajectory curvature and its graphical representation (I); an increase in the trajectory curvature of PCD microtubules can be observed. **p*<0.05.

**Figure 2**: Morphological analysis of the PCs during postnatal development (mean±SEM). A, micrographs of the PCs at different ages as seen with anti-calbindin immunofluorescence (Cb-28k; red). B-E, charts showing the quantification of the studied parameters; note that morphological alterations appear from P15 and onwards, with the soma area/size and the dendritic arbor length being the last to be significantly modified. n=4 per experimental group. **p*<0.05; ***p*<0.01.

**Figure 3**: Analysis of apoptosis along cerebellar postnatal development. A, micrograph of a Purkinje cell labeled with parvalbumin (PV, red) and TUNEL (green) in a PCD mouse. B, micrograph of an interneuron of the molecular layer labeled with NeuN (red) and TUNEL (green) in a PCD mouse. C-E, charts showing the quantification of TUNEL-positive cells in the three cerebellar layers (mean±SEM). An increase in the number of apoptotic cells can be observed from P22 and onwards in the three cerebellar layers of the PCD mice. n=4 per experimental group. **p*<0.05; ***p*<0.01.

**Figure 4**: Analysis of motor coordination and home-cage behavior along postnatal development (mean±SEM). A-B, representation and quantification of the rota-rod test. C-F, representation and quantification of the parameters analyzed in the home-cage behavior test; i.e. time for grooming, time of environmental exploratory behavior and time displacing. Note the differences derived from the *pcd* mutation for all parameters, except for displacement. n=8-9 per experimental group. **p*<0.05; ***p*<0.01.

**Figure 5**: Analysis of the novel object recognition test along postnatal development. A, schematic representation of the test and the objects employed. B-D, analysis of the percentage of time exploring familiar and novel objects at different ages for both WT and PCD mice (mean±SEM); a lack of preference for the new object is seen in PCD mice at P30. E, quantification of the number of visits to both objects at P30; no differences were observed. n=8-9 per experimental group. **p*<0.05; ***p*<0.01.

**Figure 6:** Analysis of the social preference test during postnatal development. A, diagram of test performance. B-E, charts showing quantification of the percentage of time spent exploring the chambers containing either the intruder animal or an object for both WT and PCD mice at different ages (mean±SEM). Note that PCD mice, contrary to the WT, spent the same amount of time exploring both chambers. n=8-9 per experimental group. **p*<0.05; ***p*<0.01.

**Figure 7:** Schematic representation in scale of the targeting vector used and all the possible alleles for CCP1 gene. Orange bar: genomic DNA. Black boxes: exons with their corresponding number. Green and purple arrowheads: LoxP and Flp sequences, respectively. White bar: neo cassette, with the neomycin resistance gene (white box). Blue lines: zone of sequence homology for homologous recombination, with the corresponding size in kbp. Black arrowheads: primers used for the PCR genotyping.

| **Microtubules dynamics and structure** | | |
| --- | --- | --- |
|  | **WT** | **PCD** |
| **Growing rate** | 10.62 ± 0.34 | **14.20±1.69 *** |
| **Shrinking rate** | 16.24 ± 1.67 | 21.90±2.51 |
| **Growing length** | 2.66 ± 0.10 | 2.72±0.14 |
| **Shrinking length** | 1.73 ± 0.18 | 1.81±0.15 |
| **Catastrophe frequency** | 0.09 ± 0.01 | **0.15±0.02 *** |
| **% Growing** | 92.05 ± 0.65 | 91.20±1.25 |
| **% Shrinking** | 7.67 ± 0.63 | 8.59±1.19 |
| **% Pause** | 0.28 ± 0.07 | 0.22±0.10 |
| **MTs curvature** | 3.39±0.37 | **5.58±0.74 **** |
| **MTs trajectory curvature** | 1.20±0.13 | **1.83±0.16 *** |

**Table 1**: Quantification of WT and PCD microtubule dynamics and structure. **p*<0.05; ***p*<0.01.

**Table 1**

| **PCs morphology** | | | | | | | | | | |
| --- | --- | --- | --- | --- | --- | --- | --- | --- | --- | --- |
|  | **WT** | | | | | **PCD** | | | | |
|  | **P7** | **P15** | **P17** | **P22** | **P30** | **P7** | **P15** | **P17** | **P22** | **P30** |
| **Main dendrite length (µm)** | 15.99±1.08 | 24.12±2.24 | 27.22±2.04 | 32.49±2.67 | 31.31±2.37 | 17.70±2.22 | 27.25±2.57 | **20.75±0.96*** | **25.27±0.94*** | **21.12±2.38*** |
| **Main dendrite width (µm)** | 3.61±0.25 | 3.61±0.12 | 3.68±0.09 | 2.75±0.11 | 3.24±0.32 | 3.24±0.32 | **2.81±0.16**** | **3.06±0.17*** | 2.88±0.29 | **1.97±0.31*** |
| **Dendritic arbor length (µm)** | 52.42±2.41 | 104.26±1.84 | 108.51±9.30 | 123.25±2.01 | 112.54±8.66 | 45.43±4.06 | 93.00±5.37 | 112.64±8.77 | **109.54±4.16*** | **84.59±10.74*** |
| **Soma area (µm^2^)** | 119.78±6.07 | 131.79±4.23 | 130.87±4.61 | 130.47±4.11 | 139.34±6.23 | 126.28±6.21 | 139.27±9.08 | 146.09±8.27 | **110.89±3.98**** | **71.80±13.11**** |

**Table 2**: Quantification of the PCs morphology of both WT and PCD mice. **p*<0.05; ***p*<0.01.

**Table 2**
